# Sensing Urban Transportation Events from Multi-Channel Social Signals with the Word2vec Fusion Model

**DOI:** 10.3390/s18124093

**Published:** 2018-11-22

**Authors:** Hao Lu, Kaize Shi, Yifan Zhu, Yisheng Lv, Zhendong Niu

**Affiliations:** 1 School of Computer Science & Technology, Beijing Institute of Technology, Beijing 100081, China; hao.lu@ia.ac.cn (H.L.); kzshi@bit.edu.cn (K.S.); zhuyifan@bit.edu.cn (Y.Z.); 2 The State Key Laboratory for Management and Control of Complex Systems, Institute of Automation, Chinese Academic of Science, Beijing 100190, China

**Keywords:** intelligent sensors, social transportation, multi-channel signals, event detection, word2vec-based event fusion

## Abstract

Social sensors perceive the real world through social media and online web services, which have the advantages of low cost and large coverage over traditional physical sensors. In intelligent transportation researches, sensing and analyzing such social signals provide a new path to monitor, control and optimize transportation systems. However, current research is largely focused on using single channel online social signals to extract and sense traffic information. Clearly, sensing and exploiting multi-channel social signals could effectively provide deeper understanding of traffic incidents. In this paper, we utilize cross-platform online data, i.e., Sina Weibo and News, as multi-channel social signals, then we propose a word2vec-based event fusion (WBEF) model for sensing, detecting, representing, linking and fusing urban traffic incidents. Thus, each traffic incident can be comprehensively described from multiple aspects, and finally the whole picture of unban traffic events can be obtained and visualized. The proposed WBEF architecture was trained by about 1.15 million multi-channel online data from Qingdao (a coastal city in China), and the experiments show our method surpasses the baseline model, achieving an 88.1% F_1_ score in urban traffic incident detection. The model also demonstrates its effectiveness in the open scenario test.

## 1. Introduction

Intelligent transportation systems (ITS) are highly involved in improving transportation efficiency and services [1,2]. Successful operation of ITS relies on multi-modal big data. To collect traffic data, physical sensors like inductive loops, radars, and cameras, are deployed in real world transportation systems [3,4]. However, such conventional physical sensors are expensive and provide limited coverage of transportation networks.

Today, social media and online web services provide a new path to access traffic information through the internet. Due to the widespread applications of smart devices and social networks, people can create and diffuse user generated contents (UGC) anywhere at any time. Therefore, everyone in such a network can be a social sensor to perceive the real world, which makes the collection of social signals in multiple domains possible [5,6]. Compared with traditional physical traffic sensors, social traffic sensors offer advantages of costing essentially nothing and large-scale coverage [7]. Social media data have been applied to detect traffic events, explain traffic status, analyze traffic sentiment, etc. [8,9,10].

Twitter, Facebook, Sina Weibo, News, BBS, and official transportation administration websites are the main data sources to generate social signals in transportation research [11,12,13,14,15,16,17,18,19,20,21,22]. However, current research focuses on using single channel social signals to extract traffic information. Clearly, exploiting multi-channel social signals from cross-platform online data could effectively help gain a deeper understanding of transportation events. Such multi-channel social signals provide significant opportunities, but are also accompanied by serious challenges.

In this paper, we utilize multi-channel social signals from cross-platform online data for more accurate detection and more comprehensive description of urban traffic. The main challenge is how to semantically represent and fuse the multi-channel social signals into traffic event descriptions. We firstly detect traffic events like traffic congestion, traffic incidents and traffic accidents from the News and Weibo platforms, respectively. Then we propose a word2vec-based event fusion (WBEF) model to semantically fuse the same topics detected from cross-platform data into overall traffic events. The proposed WBEF model is trained with two years’ data collected from Weibo and News platforms. We apply the proposed methods to Qingdao, a city on China’s eastern coast. Experiments demonstrate the effectiveness of the proposed methods in traffic event detection and fusion from cross-platform online data.

The main contributions of this paper are as follows: (1) We exploited cross-platform online data as multi-channel social streams to mine traffic events, which benefits from the strengths of News media’s objectivity and social media’s timeliness. (2) We proposed a word2vec-based event fusion (WBEF) model to fuse traffic events from cross-platform data semantically, and the WBEF model shows better recall and F_1_ score compared to baseline models in the experiments. (3) We deployed the proposed methods in an open scenario, obtained a complete picture of the city traffic situation, and qualitatively investigated the application efforts of proposed model. 

The rest of this paper is organized as follows: Section 2 reviews related work from three perspectives which are social transportation, topic modeling and cross-platform event detection. Section 3 presents the details of the WBEF model to detect and fuse urban traffic events from cross-platform data. Section 4 presents details of our experiments. Section 5 demonstrates a case study in Qingdao. Section 6 concludes this paper.

## 2. Related Work

Social media and online web services generate social signals via crowdsourcing technologies supported by smart mobile devices. Mining such social signals for traffic management and control has received increasing attention, and the number of publications in this field has grown quickly in recent years [23,24,25]. Typical techniques used in current social sensor-based transportation studies include natural language processing, social computing, machine learning, recommendation systems and expert systems. To our knowledge, this is the first attempt to apply the cross-platform event detection and fusion model to multi-channel social signals for urban traffic situation monitoring. In this section, we review social mediabased transportation research, and summarize topic modeling and cross-platform event fusion methods. 

### 2.1. Social Media Based Transportation Research

Social mediabased transportation research mainly falls into five categories, i.e., traffic prediction, traffic event detection, traffic sentiment, traffic reasoning, and traffic behavior analysis. Traffic prediction has a long research history. As early as the 1970s, Ahmed and Cook applied the autoregressive integrated moving average (ARIMA) model to predict short-term freeway flow [26]. Recently, social media data were used to improve traffic prediction. He et al. [27] developed a linear regression model incorporating real traffic data and Twitter data for longer-term traffic flow prediction. Ni et al. [16] showed a moderate positive correlation between subway passenger flow and Twitter post rates and proposed a hybrid model to fuse linear regression and SARIMA models to forecast subway passenger flow. Chaniotakis et al. [19] used social media data to analyze and visualize Twitter signals at both temporal and spatial levels, which showed there are indicative group mobility patterns and behavioral characteristics in urban transportation. Zeng et al. [11] studied online transportation-related topic features on the national holiday of China, from the perspectives of topic evolution analysis, opinion analysis, and geographic analysis, which could potentially help administrative sectors for traffic management.

Traffic event sensing and detection is one of the major tasks in social mediabased transportation research and applications, and many scholars have proposed inspiring models. D’Andrea, et al. [13] treated traffic event detection from Twitter as a binary classification task, which assigns traffic/non-traffic class labels to each tweet. They compared seven different classification models, such as SVM, NB, C4.5, KNN, and employed the SVM model in their proposed system since SVM achieved the best accuracy value in their tests. Gu et al. [28] defined five traffic incident categories and extracted traffic incident information on highways and arterial roads from tweet texts and they firstly used the Semi-Naive-Bayes classifier to categorize traffic incident tweets and non-traffic incident tweets, and then they trained the Supervised Latent Dirichlet Allocation classifier to identify traffic incident categories. Fu et al. [29] proposed an association rule-based keyword generation scheme to iteratively extract real time transportation incidents. Meanwhile they implemented LexRank algorithms on the complete sentence graph to rank the most influencing node, and the ranked words are regarded as the summarization of traffic incidents. Gutierrez et al. [30] presented a computational framework to detect real-time traffic events in the UK from Twitter, including tweet filtering, event type classification, name entity recognition, geo-location extraction and event tracking. Nguyen et al. [15] built a system named TrafficWatch that leveraged twitter signals and integrated them with online clustering and classification algorithms for traffic monitoring and event detection. TrafficWatch demonstrated the potential to report traffic incidents earlier than other data sources when deployed in the traffic management center of Australia. Hao et al. [31], mined the correlation between adverse weather topic heat and traffic incidents in social media, and further proposed traffic situation awareness and alerting model assisted by adverse weather data to provide information on city-level traffic situations.

### 2.2. Topic Modeling

Topic modeling tries to uncover the hidden semantic structures of different types of documents. Topic modeling technologies can obtain traffic topics from online social media and news texts. In recent years, Latent Dirichlet Allocation (LDA) [32] and its extension models have become dominant for topic modeling. Zhai et al. [33] proposed an online topic model by extending LDA to draw topics from a Dirichlet process whose base distribution is over all possible words rather than from a finite Dirichlet distribution. They also develop an online variational inference method to heuristically expand the set of words in vocabulary. Paisley et al. [34] proposed a nested hierarchical Dirichlet process for hierarchical topic modeling, and developed a stochastic variational inference algorithm for the model. The proposed method was tested on 1.8 million documents from The New York Times and 2.7 million documents from Wikipedia. 

LDA and related models are traditionally applied to long text documents like news articles, while there are increasing needs for modeling topics in short text documents like Twitter posts. Quan et al. [35] integrated topic modeling with automatic short text aggregation to alleviate the sparsity problem in short and sparse texts. Their experiments indicate the proposed scheme can extract more meaningful and interpretable topics than traditional topic models. Ramage et al. [36] developed a partially supervised LDA model which labels the content of twitter posts with four characteristics regarding substance, style, status and social relationship. The experiments indicated weighted combination of L-LDA model’s latent topic features and TF-IDF feature achieve satisfactory results for topic ranking and recommendation task. Zhao et al. [37] assumed that each tweet has been associated with a Twitter topic, and each user has its own topic distribution. Based on such a hypothesis, they proposed a twitter LDA generation process, and used Gibbs sampling to perform model inference. Experiments demonstrated the Twitter-LDA can get more meaningful topic words than standard LDA models.

### 2.3. Cross-Platform Event Detection

Multi-channel social signals processing benefits from the characteristics of multi-modality and multi-domain, which integrate different kinds of information from different sources to obtain a more comprehensive view (“big picture”) of objects compared to a single data stream. Many models with cross-platform data can produce better event prediction and detection results. Hou et al. [38] developed a cross-dependence temporal topic model to extract topics, and studied the mutual influence between news and user-generated content streams. The proposed methods were evaluated on five datasets from Sina, The New York Times and Twitter. Oghina et al. [39] used tweets from Twitter and comments from YouTube to predict IMDb movie ratings, and the best performance model could rate movies close to the observed values. Bao et al. [40] used a co-clustering model to detect emerging topics from The New York Times and Flickr which experimentally achieved effective evaluation results. Daichi et al. [41] applied a time series topic detection model to mix news and twitter streams during the London Olympic games, which detected 34 topics with a precision of 87.5%. 

Some cross-platform event detection architectures and systems have been proposed. Qian et al. [42] proposed a generic framework for social event detection, tracking and evolution analysis. They developed specific models for each task. For example, they used a boosted multi-modal supervised LDA model for social event detection, and applied an incremental topic model learning algorithm for analyzing the evolutionary processes of social events. Li et al. [43] presented the Event Knowledge from News and Opinions in Twitter (EKNOT) system which could extract summaries combining an objective description from news and opinions from tweets. They used an entity graph to link entities for an event, also used an opinion graph to get a joint summarization of an event. Wang et al. [44] proposed an event-based multi-aspect reflection mining framework to discover, link and present major events. News and tweets about a major event can complement each other to describe the event. 

## 3. Methodology

Social signals effectively connect the physical space and cyber space, which provides a new paradigm for traffic situation awareness. The main challenges that need to be faced are how to collect, process, analyze, and fuse several types of signals in social transportation. As mentioned in the related works, social transportation has attracted increasing numbers of researchers, and some frameworks/architectures were proposed for specific social transportation tasks, such as TrafficWatch [15] for traffic incident extraction, STAR-CITY [2] for traffic flow analysis and urban planning, Steds [29] for traffic event summarization, Hao’s framework [31] for weather-related traffic incidents perception. However, these architectures only leverage single social signals either from news or social media. 

Meanwhile, the frameworks for multi-modal data sensing and fusion are also getting more and more attention, with representative event detection frameworks like EKNOT [43], and the work of Wang et al. [44] and Qian et al. [42], all of which combine objective descriptions from news and opinions from tweets together, linking the event descriptions and reflection from a cross-platform with the entity graphs, finally fused into a joint summary of events. Although these frameworks recognized that fused multi-channel social signals will improve the accuracy of event detection and the diversity of event description, the entities (words representing person, location, organization, etc.) network-based event fusion methods are unable to deeply fuse the event description in a semantic way. Moreover, it is very necessary to integrate the traffic domain knowledge when applying the frameworks above to intelligent transportation systems.

To our knowledge, this is the first attempt to transfer the traffic event detection task from single channel social signals to multi-channel social signals, the main challenge being how to sense, process, analyze, link and fuse several types of signals in social transportation. To address these problems, it is necessary to integrate natural language processing, information retrieval and machine learning methods together with transportation domain knowledge to utilize the architecture. 

In this paper, by sufficiently utilizing the objectivity of news media and immediacy of social media, a word2vec-based event fusion (WBEF) model is proposed for the urban transportation event detection, which extracts topics from multi-channel social signals, semantically coupled and fused topics into cross-platform urban traffic events description. Furthermore, we develop a cross-platform traffic event detection system integrating the above methods for real world applications. 

**Figure 1:**
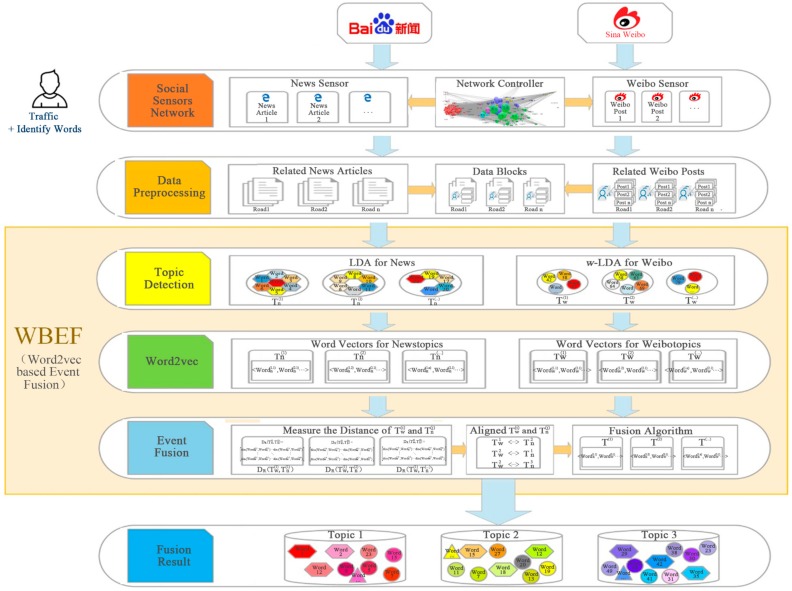
Architecture of cross-platform traffic event detection system.

The system architecture is shown in Figure 1. We choose news articles and Weibo posts as our cross-platform data sources. The multi-channel social signals from cross-platform online data are sensed through a keyword-based social sensor network configured by domain experts. The sensed webpages are filtered and decomposed into structured data, then aggregated into data blocks assigned to city roads. Traffic event topics from news articles are extracted with a news LDA model, and traffic event topics from Weibo posts are extracted with a Weibo specific model named *w*-LDA model. Furthermore, topic words are transformed into semantic representation with word vectors, and then the cross-platform traffic events can be fused based on the topic distance matrix semantically. The expressions and descriptions of variables in the following algorithms or sub-models are summarized in Appendix A.

### 3.1. Social Sensors Network

**Figure 2:**
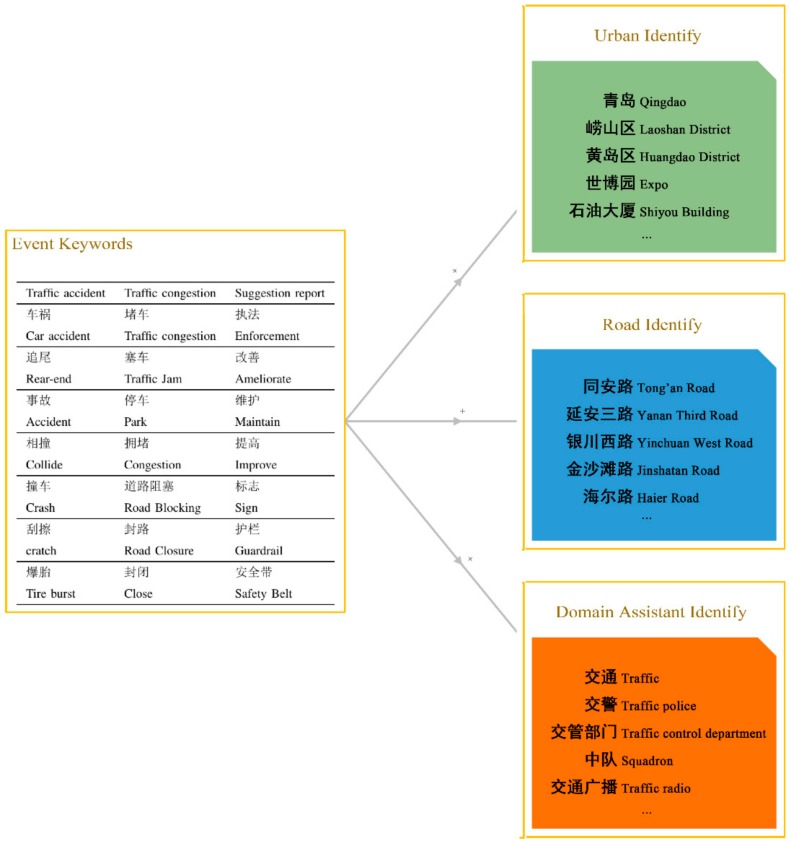
Traffic keywords for social sensors.

The social signals can be typically sensed by two approaches. The first approach is using API services provided by the platform to parse the XML or JSON file, such as Twitter and Weibo. The second approach is by deploying a web crawler that periodically monitors keywords, accounts and URL lists. In our system, we take both approaches according to the availability and limits of service providers. By consulting experts in traffic administrative agencies, the traffic keywords are grouped into four categories which are traffic event keywords, urban identity keywords, road identity keywords and domain assistant identity keywords, respectively. Specifically, traffic event keywords are mainly exploited to describe three types of traffic events, which are traffic accidents, traffic jams and traffic suggestions. The identity keywords (Figure 2) describe corresponding cities, roads, and domain assistants. All above traffic keywords are treated as search seeds that are feeding into the social sensor network. 

The News sensor perceived the latest news data according one specific keyword from the News search engine. Correspondingly, the Weibo sensor also perceived latest Weibo data according one specific keyword from the Weibo search engine. The News and Weibo sensors were deployed to continuously monitor the data according to a keywords list, and the links of different sensors were built if both sensors acquired the same News or Weibo article. Then, all the sensors and corresponding links eventually formed a social sensors network, which dynamically adapted to perceiving multi-channel social signals. 

The keywords-based social sensors network consists of a crawler, page parser, duplicated URLs filter, etc. [45]. Particularly, to efficiently utilize the network bandwidth and computation resources, the social sensors network has a keywords priority adaptor, which dynamically sorts the keyword query priorities according to the value of importance ranking for each node in the keyword network.

**Figure 3:**
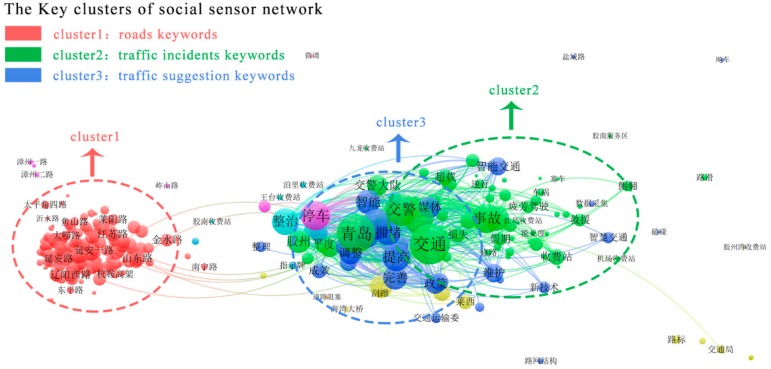
The keywords-based social sensors network.

The keywords-based social sensors network is visualized in Figure 3. The nodes denote traffic keywords. The size of each nodes represents the number of webpages that are collected regarding the node’s keywords. The edges are constructed by calculating the co-occurrence of keyword pairs in the same document. All the search keywords are aggregated into network clusters, the main clusters include the roads cluster, the traffic incidents cluster and traffic suggestion keywords. Since the experiment is based on a Chinese corpus, here we only show the social sensors network with Chinese nodes and annotate the important information in English. The social sensors network can also be analyzed by a social network algorithm, such as ranking the betweenness, closeness and degree. 

### 3.2. Data Preprocessing

Data preprocessing is a fundamental step for traffic event sensing from cross-platform media because raw data are unstructured and full of noise. The major preprocessing steps include meta-data extraction, noise filtering, word segmentation and data blocks aggregation:

*Noise filtering:* The URL links, paragraph marks, emoji, etc. in the texts are regarded as noise information, which negatively influence the accuracy and efficiency of word segmentation, information processing and model training. To solve the problems, we define regular expressions to represent various noise patterns, and then use the regex to search and delete noises in the text. Meanwhile, either too short or too long texts are removed.

*Meta-data extraction*: we deployed Dom wrapper and XPath parsers to extract the title, post time and other meta-data like authors, review number and repost number, etc. from the sensed web page cache. Then the meta-data information is stored in databases. 

*Word segmentation*: The space is a natural word delimiter in English texts, however, there is no such equivalent in Chinese, so word segmentation is needed for Chinese NLP tasks. The Language Technology Platform (LTP) [46] was deploy to segment words, remove punctuation and stop words, and tagging the road entity with customized dictionary. The customized dictionary for LTP includes all the keywords we used for the social sensors. 

*Data blocks aggregation*: The sensed multi-channel social signals are aggregated into data blocks, which are defined as a dataset containing cross-platform online data related to every urban road. With the LTP’s entity recognition tools, the road entity of every News article or Weibo post is extracted, then the cross-platform data are aggregated into data blocks corresponding to the road entity. Moreover, when news articles or Weibo posts contain multiple road entities they will be assigned to each corresponding data block separately. Each data block was fed into the event detection and fusion models iteratively.

### 3.3. Word2vec Based Event Fusion Model

News articles usually describe a traffic event with relatively standard language, while Twitter and Weibo may have posts expressing opinions/comments/discussions on the same traffic event [47,48]. Matching news articles and Twitter/Weibo posts on the same transportation topic can give a more comprehensive description of a traffic event. Consequently, we propose to use LDA-based models to extract topics in each respective source channels and link them together with the WBEF model.

#### 3.3.1. Transportation Events Detection from News and Weibo 

As described in the literature review section, the LDA and its variants have been widely applied for event detection. To group transportation topics and find topic words from News, we use the standard LDA model for event detection. While for Weibo, we detect traffic events with a *w*-LDA model which is described in details as follows:

*w-LDA model*: The *w*-LDA model is based on the USER scheme which achieves good performances in Twitter classification [49]. The process is described as follows:

a) Combine all training messages generated by the same user into user profiles;
b) Train the *w*-LDA model with training user profiles;
c) Aggregate all testing messages generated by the same user into testing user profiles;
d) Use the trained *w*-LDA model to infer a topic mixture.

The aggregated user profiles can be viewed as a random mixture distribution over latent topics, where each topic is characterized by a distribution over words. Both distributions are assumed to have a sparse Dirichlet prior. Suppose the corpus consists of *T* Weibo posts and *U* users that are aggregated into *P* user profiles, each user profile *p* contains *N_p_* words. The total number of topics denoted as *K*, the unique words in vocabulary are denoted as *V*. 

There are five latent variables and one observable variable, where latent variable α is a *K*-dimensional vector giving uniform prior weight for all topics in a user profile *p*, latent variable β is a *V*-dimensional vector with uniform prior weights for all words in a topic *k*, latent variable $z_{i}$ is the topic for *i*-th word in user profile *p* and observable variable $w_{i}$ is the specific word, latent variable $\varphi_{z}$ is a *V*-dimensional vector representing the Dirichlet topic distribution for user profiles, latent variable $\vartheta_{p}$ is a *K*-dimensional vector representing the Dirichlet word distribution for topics. The variable $z_{i}\;$ and $w_{i}$ are drawn from multinomial distributions. The generative process can be seen in Algorithm 1.



**Algorithm 1. *w*-LDA Generation Process**
**Input:*****K***, ***U***, ***T***, α, β
**Output:**
$\varphi_{z}$
***, $\vartheta_{p}$, uProfileSet***
//*Step 1: User profiles data generation*For each author *u* = *1…U*  Traverse all the Weibo posts ***t*** = *1…T*;Aggregate the posts generated by author *u* into *users’ profile s **uProfileSet(p)***
*Return **uProfileSet***
//*Step 2: Topics generation*For each topic *z* = *1…K*  Draw $\varphi_{z}$ ~ Dirichlet (β)// sample mix components for topic-word
*//Step 3: Topic words generation*
For each user profile *p* = *1…P*  Draw $\vartheta_{p}$ ~ Dirichlet (α)// sample mix components for user profile-topic  For each word in generated user profile *p*, *i* = *1…N_p_*    Draw $z_{i}$ ~ Multinomial ($\vartheta_{p}$) // Sample topic $z_{i}$ for user profile *p*    Draw $w_{i}$~ Multinomial ($\varphi_{z_{i}}$) //Sample word $w_{i}$ for topic $z_{i}$Return $\varphi_{z}$ and $\vartheta_{p}$



The *w*-LDA model focuses on finding out topics for each user profile, and we use collapsed Gibbs sampling to inference the final goal that is to approximate the distribution of $P\;\left(z_{i}\;=\;j|\;Z_{-i},w_{i},p_{i}\right)$, which is:

(1) $$P\left(z_{i}\;=\;j|\;Z_{-i},w_{i},p_{i}\right)\propto \frac{n_{-i,j}^{\left(w_{i}\right)}\;+\;\beta }{\sum_{i=1}^{v}n_{i,j}^{\left(w_{i}\right)}\;+\;V\beta }\;\times \;\frac{n_{-i,j}^{\left(p_{i}\right)}\;+\;\alpha }{\sum_{j=1}^{K}n_{i,j}^{\left(p_{i}\right)}\;+\;K\alpha \;}$$

where $P\;\left(z_{i}\;=\;j|\;Z_{-i},w_{i},p_{i}\right)$ denotes as the probability that word $w_{i}$ is assigned to topic j, $Z_{-i}$ represents topic j assigned to all other words, $w_{i}$ represents the *i*-th word in the vocabulary, $p_{i}$ represents the user profile containing the word $w_{i}$. Then $n_{-i,j}^{\left(w_{i}\right)}$ is the number of times all word $W_{-i}$ assigned to the topic j excluding the current word $w_{i}$, $\sum_{i=1}^{v}n_{i,j}^{\left(w_{i}\right)}$ calculates the total number of all words W assigned to the topic j. $n_{-i,j}^{\left(p_{i}\right)}$ is the number of times topic j is assigned to words in the user profile $P_{-i}$ excluding the current user profile $p_{i}$, $\sum_{j=1}^{K}n_{i,j}^{\left(p_{i}\right)}$ calculates the total number of all words in user profile $p_{i}$.

$\varphi_{z}$ represents the predictive distributions of words in topic *z*, $\vartheta_{p}$ represents the predictive distributions of topics in user profile p. For any obtained sample we can estimate $\varphi_{z}^{\left(j\right)}$ and $\vartheta_{p}^{\left(j\right)}$ by:

(2) $$\varphi_{z}^{\left(j\right)}\;=\;\frac{n_{i,j}^{\left(w_{i}\right)}\;+\;\beta }{\sum_{i=1}^{v}n_{i,j}^{\left(w_{i}\right)}\;+\;V\beta },\;\vartheta_{p}^{\left(j\right)}\;=\;\frac{n_{i,j}^{\left(p_{i}\right)}\;+\;\alpha }{\sum_{j=1}^{K}n_{i,j}^{\left(p_{i}\right)}\;+\;K\alpha }$$

where $n_{i,j}^{\left(w_{i}\right)}$ is number of times that word $w_{i}$ has been assigned to topic *j*, $n_{i,j}^{\left(p_{i}\right)}$ is the number of times topic *j* has been assigned to words in user profile $p_{i}$.

#### 3.3.2. Transportation Events Representation 

After detecting topics from News articles and Weibo posts separately, we obtain the bags of words as event descriptions. The widely used one hot vector presentation for each topic word is unable to calculate the semantic similarity of topic words from different platforms efficiently, therefore, we trained the transportation word embeddings to represent topic words and calculated the semantic similarity between topic pairs. Finally, we linked and fused the topic pairs into event descriptions. Traditionally, in natural language processing each word is represented as a one-hot vector which is 1 at the position associated with the word an 0 at other positions. Clearly, the one-hot representation cannot capture any information about the semantic similarity between words. Moreover, the one-hot vector is high-dimensional and sparse. Recently, word embedding is proposed to represent each word in a continuous vector space and encode many semantic patterns [50,51]. Word embedding was firstly presented by Bengio et al. in [52], and implemented by Mikolov et al. in word2vec [53]. Since then word2vec has gained popularity for natural language processing [54], question and answer systems [55], information retrieval [56], recommending systems [57,58], sentiment analysis [59] etc.

**Figure 4:**
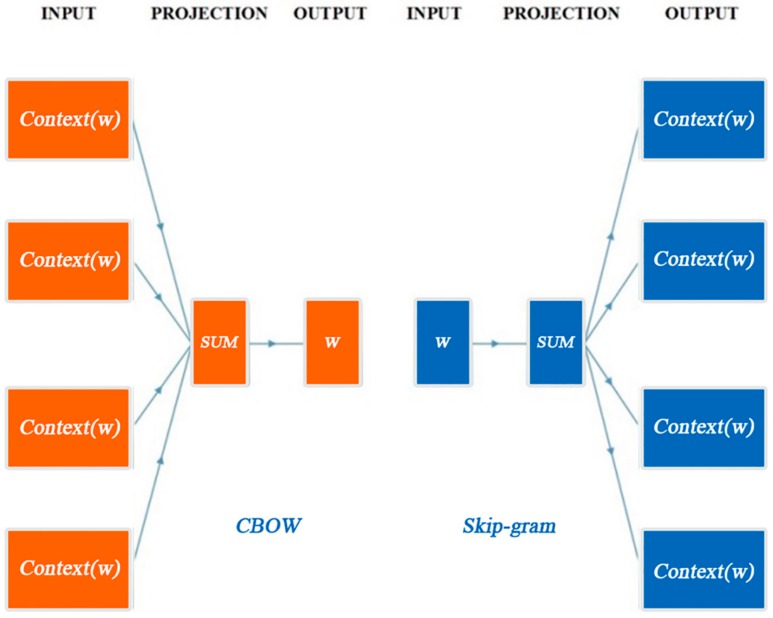
Model structure of CBOW and Skip-gram.

The basic idea of word2vec is to combine a word and its contextual information together, and encode them into a low-dimensional vector. Words with similar contexts in the corpus are located in close proximity to each other in the representation space. Word embedding can be trained either by the Continuous Bag of Words (CBOW) model or the Skip-gram model. Both of them are neural networks which map word(s) to the target variable which is also a word(s), and the learned weights are word embedding representations. Specifically, the CBOW model is learning to predict the word by the context words, in contrast the skip-gram model is learning to predict the context words from the current word. The simplified structure of the two models is shown in Figure 4. Herein, we choose the CBOW model for training transportation word embedding. 

However, computing probabilities in softmax layer is the most resource consuming phase when training CBOW models, since it requires summing over all words in the large vocabulary. Therefore, we use the Negative-Sampling [60] method to approximate the softmax layer in the CBOW model, which moves the embedding toward the neighbor words and away from the noise words. In this paper, the noise words are sampled from vocabulary according to their weighted the 3/4 power of unigram probability. 

#### 3.3.3. Transportation Events Fusion

Transportation topic clusters and corresponding topic words associated with each cluster can be obtained through the LDA model for news articles and *w*-LDA model for Weibo. The topic word clusters for Weibo and News are denoted as $T_{w}^{\left(i=1\ldots K\right)}$ and $T_{n}^{\left(j=1\ldots K\right)}$, respectively, where *K* is the total number of topic clusters. Each topic has a set of words, and we denote words in the given topic cluster *i* and *j*, as $WE_{w}^{\left(m=1\ldots M,i\right)}$ and $WE_{n}^{\left(n=1\ldots N,j\right)}$ separately, where *M*, *N* are the total number of words in the given topic clusters.

- *Events similarity measure:*

Meanwhile, each word is represented as a 200-dimensional continuous vector. Based on the semantic representation, we define words distance $dis_{R}\left(WE_{w}^{\left(m,i\right)},WE_{n}^{\left(n,j\right)}\right)$ to measure the similarity between words in the given topic cluster $T_{w}^{\left(i\right)}$ and $T_{n}^{\left(j\right)}$, where R = {Cosine distance, Euclidean distance, Manhattan distance, Chebyshev distance, Weighted Euclidean distance}.

The formulas of the above distances are listed in Table 1. Furthermore, a topic distance matrix $D_{R}\left(\;T_{w}^{\left(i\right)},\;T_{n}^{\left(j\right)}\right)$ can be defined as follows to measure the distance between $T_{w}^{\left(i\right)}\;\mathrm{and}\;T_{n}^{\left(j\right)}$:

(3) $$D_{R}\left(\;T_{w}^{\left(i\right)},\;T_{n}^{\left(j\right)}\right)\;=\;\left[\begin{array}{ccc} dis_{R}\left(WE_{w}^{\left(1,i\right)},WE_{n}^{\left(1,j\right)}\right) & \cdots & dis_{R}\left(WE_{w}^{\left(1,i\right)},WE_{n}^{\left(N,j\right)}\right)\\ \vdots & \ddots & \vdots \\ dis_{R}\left(WE_{w}^{\left(M,i\right)},WE_{n}^{\left(1,j\right)}\right) & \cdots & dis_{R}\left(WE_{w}^{\left(M,i\right)},WE_{n}^{\left(N,j\right)}\right) \end{array}\right]\;$$

- *Events alignment:*

Topic alignment is to align topics detected from different platforms. The topics from different platforms are latent variables, thus we do not know each topic labels like traffic jam and traffic accidents. Hence, we need to align the topic clusters detected from the News platform and Weibo platform into topic pairs, which will support the multi-view descriptions in the fusion step.

From bottom up, we choose a $WE_{w}^{\left(m,i\right)}$ in $T_{w}^{\left(i\right)}$ to calculate the similarity with each word in $T_{n}^{\left(j\right)}$, then find the most similar or closest $WE_{w}^{\left(n,j\right)}$ in $T_{n}^{\left(j\right)}$. Based the closest word pairs, the shortest distance between $T_{w}^{\left(i\right)}\;\mathrm{and}\;T_{n}^{\left(j\right)}$ denoted as $\underset{m,n=1\ldots N}{\min}dis_{R}\left(WE_{w}^{\left(m,i\right)},WE_{n}^{\left(n,j\right)}\right)$, the average shortest distance between $T_{w}^{\left(i\right)}\;\mathrm{and}\;T_{n}^{\left(j\right)}$ denoted as $\mu_{R}\left(T_{w}^{\left(i\right)},T_{n}^{\left(j\right)}\right)$, the normalized average shortest distance $\mu_{R}^{*}\left(T_{w}^{\left(i\right)},T_{n}^{\left(j\right)}\right)$, and the standard deviation $\sigma_{R}^{*}\left(T_{w}^{\left(i\right)},T_{n}^{\left(j\right)}\right)$ can be derived:

(4) $$\;\mu_{R}\left(T_{w}^{\left(i\right)},T_{n}^{\left(j\right)}\right)\;=\;\frac{\sum_{m=1}^{M}\underset{n=1\ldots N,n\neq m}{\min}dis_{R}\left(WE_{w}^{\left(m,i\right)},WE_{n}^{\left(n,j\right)}\right)}{M}\;$$

Next, we select a topic $T_{w}^{\left(i\right)}$, calculate the normalized average distance $\mu_{*}^{R}\left(T_{w}^{\left(i\right)},T_{n}^{\left(j\right)}\right)$ between topic $T_{w}^{\left(i\right)}$ and topics in $T_{n}^{\left(j\right)}$,

(5) $$\mu_{R}^{*}\left(T_{w}^{\left(i\right)},T_{n}^{\left(j\right)}\right)\;=\;\frac{\mu_{R}\left(T_{w}^{\left(i\right)},T_{n}^{\left(j\right)}\right)-\underset{i=1\ldots k,j=1\ldots k}{\min}\mu_{R}\left(T_{w}^{\left(i\right)},T_{n}^{\left(j\right)}\right)}{\underset{i=1\ldots k,j=1\ldots k}{\max}\mu_{R}\left(T_{w}^{\left(i\right)},T_{n}^{\left(j\right)}\right)-\underset{i=1\ldots k,j=1\ldots k}{\min}\mu_{R}\left(T_{w}^{\left(i\right)},T_{n}^{\left(j\right)}\right)}$$

Then, the topic $T_{w}^{\left(i\right)}$ was aligned with the most similar topic $T_{n}^{\left(j\right)}$ through calculating $\underset{j=1\ldots k}{argmin}\;\mu_{R}^{*}\left(T_{w}^{\left(i\right)},T_{n}^{\left(j\right)}\right)$. Finally, the index pair (i, j) of aligned topics was returned.

- *Events fusion:*

After aligning the cross-platform traffic topics for each urban road, we can fuse the aligned topic pairs into a unified event description. 

First, the anomalous words were removed. We scan the words list $WE_{w}^{\left(*,i\right)}$ in $T_{w}^{\left(i\right)}$, and calculate the shortest distance $\underset{n=1\ldots N,n\neq m}{\min}dis_{R}\left(WE_{w}^{\left(m,i\right)},WE_{n}^{\left(n,j\right)}\right)$ from current word $WE_{w}^{\left(m,i\right)}$ to the aligned topic words $WE_{n}^{\left(*,j\right)}$. If the shortest distance falls outside the region of three standard deviations $\sigma_{R}^{*}\left(T_{w}^{\left(i\right)},T_{n}^{\left(j\right)}\right)$, $WE_{w}^{\left(m,i\right)}$ will be regard as an anomalous word:

(6) $$\sigma_{R}^{*}\left(T_{w}^{\left(i\right)},T_{n}^{\left(j\right)}\right)\;=\;\sqrt{\frac{\sum_{i=1}^{K}{\left(\mu_{R}^{*}\left(T_{w}^{\left(i\right)},T_{n}^{\left(j\right)}\right)\;-\;\frac{1}{K}\sum_{j=1}^{K}\mu_{R}^{*}\left(T_{w}^{\left(i\right)},T_{n}^{\left(j\right)}\right)\right)}^{2}}{K}}$$

Second, the topic words from cross-platform were fused. If the shortest distance falls inside the region of one standard deviation $\sigma_{R}^{*}\left(T_{w}^{\left(i\right)},T_{n}^{\left(j\right)}\right)$, the current word will be replaced with candidate $WE_{n}^{\left(n,j\right)}$ for a more objective and formal event description. The detailed algorithm for event fusion is shown in Algorithm 2.



**Algorithm 2. Transportation Events Alignment and Fusion**
**Input:**$T_{w}$**,**$T_{n}$**,**$WE_{weibo}$, $WE_{news}$
**Output: *AlignedTopicsList, FusionEventMatrix***
*//Step 1: aligning the topic clusters in*$T_{w}$*and*$T_{n}$, *return AlignedTopicsList* For each $T_{w}^{\left(i\right)}$ in $T_{w}$  WeiboIndex = *i*  For each $T_{n}^{\left(j\right)}$ in $T_{n}$   If $\mu_{R}^{*}\left(T_{w}^{\left(i\right)},T_{n}^{\left(j\right)}\right)<\mathrm{minTopicDis}$   minTopicDis$\;=\;\mu_{R}^{*}\left(T_{w}^{\left(i\right)},T_{n}^{\left(j\right)}\right)$   NewsAlignedIndex = *j* End for // *Find the closest topic cluster* Append (WeiboIndex, NewsAlignedIndex) to AlignedTopicsList End for // *couple closet the topic clusters into pairs*//*Step 2: fusing the words in the cross-paired topics, return FusionEventMatrix* For each index pair in AlignedTopicsList  *i* = WeiboIndex;  *j* = NewsAlignedIndex  LB_n_ = $\mu_{R}^{*}\left(T_{w}^{\left(i\right)},T_{n}^{\left(j\right)}\right)$$\;-\;\sigma_{R}^{*}\left(T_{w}^{\left(i\right)},T_{n}^{\left(j\right)}\right)$   UB_w_ = $\mu_{R}^{*}\left(T_{w}^{\left(i\right)},T_{n}^{\left(j\right)}\right)$ + $\sigma_{R}^{*}\left(T_{w}^{\left(i\right)},T_{n}^{\left(j\right)}\right)$ // *calculate the low boundary and up boundary for news words*  LB_w_ = $\mu_{R}^{*}\left(T_{w}^{\left(i\right)},T_{n}^{\left(j\right)}\right)$ − $3\sigma_{R}^{*}\left(T_{w}^{\left(i\right)},T_{n}^{\left(j\right)}\right)$  UB_w_ = $\mu_{R}^{*}\left(T_{w}^{\left(i\right)},T_{n}^{\left(j\right)}\right)$ + $3\sigma_{R}^{*}\left(T_{w}^{\left(i\right)},T_{n}^{\left(j\right)}\right)$ // *calculate the low boundary and up boundary for weibo words*  For each $WE_{w}^{\left(m,i\right)}$ in $T_{w}^{\left(i\right)}$   For each $WE_{n}^{\left(n,j\right)}$ in $T_{n}^{\left(j\right)}$    If $dis_{R}\left(WE_{w}^{\left(m,i\right)},WE_{n}^{\left(n,j\right)}\right)\;<\;\mathrm{minWordDis}$    minWordDis = $dis_{R}\left(WE_{w}^{\left(m,i\right)},WE_{n}^{\left(n,j\right)}\right)$    minWordIndex = *n*   End for // *Find the shortest word embedding distance in coupled clusters*   If LB_w_ < minWordDis < UB_w_ // *remove anomaly word in Weibo cluster*    If LB_n_ < minWordDis < UB_n_     Append $WE_{n}^{\left(minWordIndex,j\right)}$ to FusionEventList // *append words in News cluster*    Else    Append $WE_{w}^{\left(m,i\right)}$ to FusionEventList // *append words in Weibo cluster*  End for  Append FusionEventList to FusionEventMatrix End for


## 4. Experiments 

### 4.1. Data Description

The proposed methodology was applied to detect and fuse urban traffic events in Qingdao (a coastal city of China) from cross-platform social signals. Hence, first we used a social sensors network with 337 traffic keywords to collect data from News and Weibo which related to Qingdao transportation.

After obtaining the raw webpages, we removed the News articles which content length was greater or less than 90% of articles, and also removed the Weibo posts with a number of words of less than 5. Meanwhile, considering there are lots of social bots or online spammers in Weibo [61,62,63,64], we only retained the authors that published less than 10 articles in one day.

Next, we removed the punctuation, paragraph symbols, and noise patterns in the Weibo posts and news articles. Then we segmented texts into words, removed stop words, and tagged the words representing city roads with the LTP toolkit. After consulting the local transportation agency, we annotated 132 main roads in Qingdao, and aggregated the preprocessed texts into road data blocks with the criteria we mentioned in Section 3.

Finally, the multi-channel Qingdao transportation dataset from 1 August 2015 to 4 August 2017 was built. The dataset has about 1.15 million texts in total, including 301,684 News articles and 839,587 Weibo posts. The dataset was divided into training dataset, testing dataset and case study dataset, as shown in Table 2. In the following section, we separately used the training dataset to learning WBEF model parameters, the testing dataset to evaluate model’s performance, and the case study dataset to discuss the practical application effect of proposed model in open scenario. 

### 4.2. WBEF Model Verification

In this section, we first describe the implementation details of WBEF model, show the performance of each sub-model, evaluate the entire performance of the overall WBEF model, and compare the WBEF model with the baseline model. 

#### 4.2.1. Transportation Word Embedding

We utilized the CBOW algorithm to train transportation word embedding (200 dimensions). The window size is 5, which indicates the maximum distance between the current and predicted word within a sentence. The number of training epochs over the dataset is 10. The negative sampling algorithm is used to approximate the parameters’ gradient, and the number of noise words drawn for current word is 5. 

To evaluate transportation word embedding, we selected the most representative words in transportation events (i.e., traffic congestion, traffic accident), while searching for the most similar word embedding semantically. Table 3 gives top five most similar words for traffic congestion and traffic accident. The results show that our transportation word vectors contain essential semantic information.

#### 4.2.2. Transportation Event Detection 

**Figure 5:**
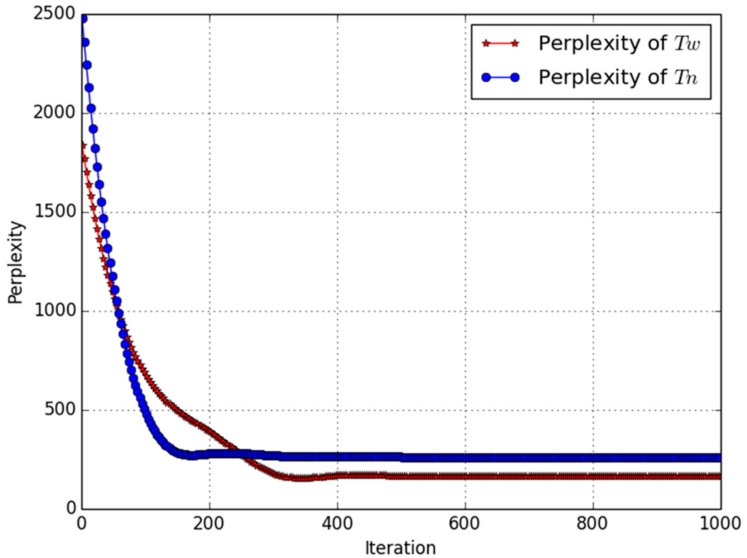
Average Perplexity of LDA for News and *w*-LDA for Weibo posts.

During the experiments, we tried different K values (K = 1–5) for aggregating topic clusters, and when K = 3 we get more meaningful word clusters for every topic. We used the perplexity value to evaluate the LDA and *w*-LDA models, respectively. Since there were 132 data blocks that represent multi-channel social signal sensed from the main roads in Qingdao, so the LDA and *w*-LDA model were deployed on every data block for topic generation. Hence, the LDA and *w*-LDA model parameters were determined by the average perplexity of topic model on multiple data blocks as shown in Figure 5, the average perplexity of *w*-LDA converge to the value of 167 after 347 training iterations, the average perplexity of LDA converge to the value of 279 after 182 training iterations. Considering the model generalization on different roads in Qingdao, we choose the topic model which perplexity value was closest to the average perplexity. 

In order to validate the LDA and *w*-LDA topic models, we selected the three largest data blocks in the testing dataset to extract topics for the corresponding road. Herein, to intuitively show the results, we list the words in the largest cluster of ***T_w_*** and the words in the aligned cluster of ***T_n_*** for each road (Table 4).

#### 4.2.3. Distance Metrics for Transportation Events Fusion

We tested different distance metrics for fusing traffic topics, and chose the proper distance metric for semantic event fusion. The top 10 largest road data blocks in the testing dataset were selected, and the topics were extracted from each data block with the LDA and *w*-LDA models, respectively. The words in the largest topic cluster $T_{w}^{\left(i\right)}$ and the corresponding aligned cluster $T_{n}^{\left(j\right)}$ were considered as the fusion corpus for distance comparison experiments. 

For each distance measure methods we computed the average and standard deviation of topic distances $D_{R}\left(\;T_{w}^{\left(i\right)},\;T_{n}^{\left(j\right)}\right)$ on different data blocks, expressed as $\mu_{R}\left(T_{w}^{\left(i\right)},T_{n}^{\left(j\right)}\right)$ and $\sigma_{R}^{*}\left(T_{w}^{\left(i\right)},T_{n}^{\left(j\right)}\right)$. For the model robustness, we chose the distance has the minimum standard deviation on multiple roads, which can be obtained through $\arg\underset{R}{\min}\sigma_{R}^{*}\left(T_{w}^{\left(i\right)},T_{n}^{\left(j\right)}\right)$. The results in Table 5 show that the most stable distance measure is the cosine distance for word embedding presentation. 

### 4.3. Overall Performance

To evaluate the overall performance of the proposed model, we selected 10 roads in Qingdao with the most frequently occurring traffic events, checked the content of News and Weibo articles in every road data block, and manually annotated the traffic events including traffic accidents, traffic jams and traffic complements in the testing dataset. By consulting a traffic domain expert from the Qingdao Transportation Committee, the traffic events annotation results which consisted of 27 traffic accidents, 47 traffic jams, and 13 traffic complaints in testing dataset were finally confirmed.

**Figure 6:**
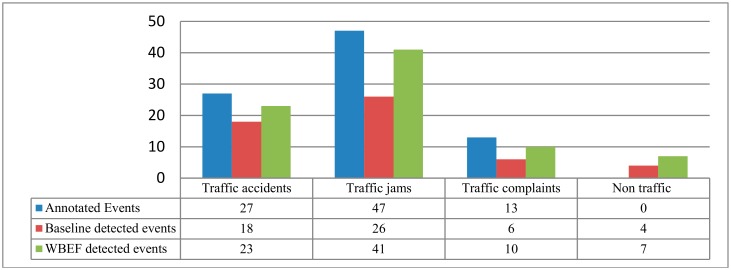
Comparison of detected and annotated number of traffic events.

Moreover, the dataset was built with keywords-based social sensors, thus there will be non-traffic News and Weibo articles which also contain traffic keywords, such as traffic products release, traffic safety lectures, and civilized traffic initiative, etc. Hence, if the events were widespread in social media or news, the model may also falsely flag these non-traffic events. In that case, the precision, recall and F_1_ Score were adopted to evaluate the model performance. The proposed model was applied to detect and fuse traffic events on the 10 roads during one week. Meanwhile, we selected the “standard LDA + keywords matching” approach as the baseline model. Experimental results are given in Figure 6 and Table 6. Although the precision value of our proposed model (91.4%) is less than baseline model (92.6%), the recall value (85.1%) and F_1_ score (88.1%) is much better than baseline. Overall the WBEF model surpassed the baseline model. 

The experimental results shows that the baseline model “standard LDA + keywords matching” is unable to effectively process short content in Weibo, and also lacks semantic meaning when the different word styles from cross-platform media are fused, so fewer traffic events have been sensed. However, the event words both exactly occurred in News articles and Weibo posts imply the traffic events have been confirmed by both officials and the masses, which leads to higher accuracy of the baseline model. Compared to the baseline model, the WBEF model grouped the short messages into user profiles, then processed and clustered user-central context through the *w*-LDA scheme, hence traffic topics in Weibo can be detected more effectively. Meanwhile, with the WBEF model, the detected cross-platform topics can be embedded with semantics, which guarantees topic similarity calculation and event fusion, so more traffic events can be detected, resulting in much higher recall value, which effectively solves the problem of missing detection in the baseline model. However, the noise information in the dataset that is collected by the keywords-based social network sensors, also caused bias in the processing and fusion step, which leads to slightly lower precision. 

Quantitatively evaluation, the F_1_ score of WBEF model exceeded the baseline model by nearly 17 percentage points, which means the proposed model is much superior to the baseline model in sensing and detecting traffic events from multi-channel social signals. In the next section, we will choose one case, qualitatively study and discuss the traffic event detection effects of WBEF approaches in open scenario. 

## 5. Case Study in Application Scenario

In this section, we chose the traffic situation in Qingdao on 4 August 2017 as one case, sensed multi-channel social transportation signals and deployed the WBEF model in the open scenario. As shown in Table 2, the case study dataset contains about one thousand articles. The WBEF model successfully identified 11 traffic events, missed two traffic events and false alarmed one traffic event, finally achieving 91.6% precision, 84.6% recall and an 87.9% F_1_ score.

**Figure 7:**
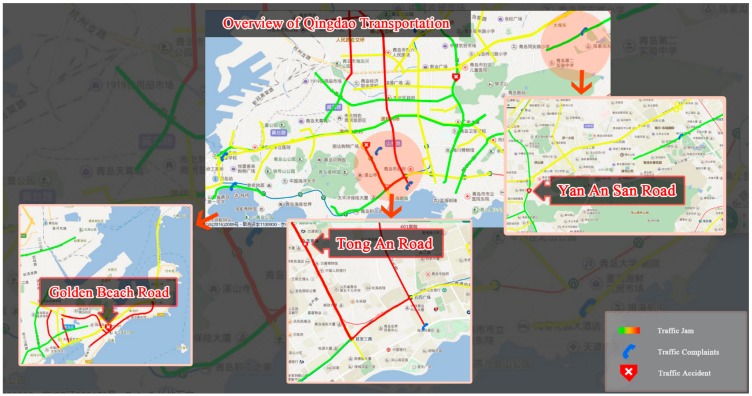
Overview of Qingdao transportation on 4 August 2017.

All the traffic events were mapped to the city roads, as shown in Figure 7, and the overall urban transportation situation can be visualized, which will intuitively support traffic management and traffic plan decisions. Meanwhile, in order to qualitatively investigate the practical usability and effectiveness of the proposed model, we chose the Top 3 hottest events (largest clusters) that occurred on different roads, and imported the corresponding road block data into the baseline and WBEF models. Although both models successfully detected Top 3 hottest events, the baseline model generated fewer words with less semantic. In contrast, the WBEF model presented more comprehensible and understandable words description of transportation events.

Furthermore, we investigated event detail and corresponding causal factors. To clearly demonstrate event words, we manually categorized and tagged event words into target road words, relevant location words, traffic words, reason words, and other words (shown in Table 7). We also double-checked News and Weibo data related to the event and got insights into the event details. Three target roads with detected traffic events are detailed in the following.

Gold Beach Road (金沙滩路): on this road, event fusion words included traffic words “parking place (停车场)”, “traffic jam (交通拥堵)”, “slow down (慢行)”, “traffic broadcast (交通广播)”, social activity words like “beer festival (啤酒节)”, “opening ceremony (开幕式)”, and famous star words like “Xiaoming Huang (黄晓明)”. After referring to Weibo and News data associated to this event, we found that the annual Qingdao International Beer Festival was opening on the square of Qingdao Beer City, where the film star Xiaoming Huang and other famous stars presented a show for the celebration. In addition, the ceremony was opening at the evening rush hour (7 p.m.) which caused a heavy traffic jam on Gold Beach Road.

Tong An Road (同安路): with the proposed methods, we observed the location words “GuoXin stadium (国信体育场)” and traffic irrelevant words “Mayday (五月天, a singer group)”, “concert (演唱会)” in the event description. However, there was no words about traffic jams or accidents, but only traffic words like “detour (绕行)”, “control (管制)”, “dispatch (调流)”, “plan (预案)” etc. We further checked the relevant Weibo and News data, then found that a concert would be held on the next day, so the traffic agency was providing early alerts about the traffic situation on the road, releasing the traffic control notice through social media and news. Massive fans and audiences forwarded and disseminated these posts.

Yan An San Road (延安三路): The event words include traffic words like “traffic jam (交通拥堵)”, “evacuation (疏散), and also fire emergency words like “fires (火情)”, “fireman (火警)”. Associated with the location word “Petroleum Building (石油大厦)”, we inferred there was a traffic jam on “Yan’An San Road (延安三路)” which was caused by a fire emergency. After further checked, the real fact was consistent with the detected results from cross-platform media. The elevator in the Petroleum Building caught fire at 7 p.m. Lots of citizens posted the live fire and traffic situation, gave notices and conveyed safety messages to their family. The official announcement also reported the event through news and social media.

## 6. Conclusions and Future Work

In Intelligent transportation systems, the method of analyzing social signals (social transportation) offers advantages of low cost and large coverage over traditional methods which depend on physical sensors. In this paper, we addressed the challenges of cross-platform traffic event detection when shifting the social signals from a single channel to multiple channels. The WBEF model for urban transportation event detection has been proposed, which benefits from comprehensive social signals, domain knowledge and semantic representation. The model was trained with about 1.15 million News and Weibo data from the past 2 years, and deployed to assess the traffic situation in Qingdao. Experiments show that the sub-models of WBEF achieved the expected performance, and the overall performance of WBEF is much superior to the baseline model. Moreover, from the case study in the open scenario, the accuracy and robustness of WBEF have been further demonstrated.

Further investigation can be conducted: firstly, more social signal sources can be involved into the WBEF model, such as Instagram, Facebook, Quora, etc., which will make traffic incident detection more accurate and comprehensive. Secondly, powerful deep learning methods have highly potentials to improve accuracy and robustness for cross-platform event detection. Thirdly, the WBEF model can be extended to heterogeneous recommending system [65,66], which will achieve more personalized and accurate information services in transportation domain. Furthermore, social sensors combine with physical sensors (Cyber-Physical-Social-System, CPSS) will lead a novel way to monitor, control, and optimize intelligent transportation systems.

## Figures and Tables

**Table 1:** Formulas of the words vector Distance.

R=	Formula
Euclidean Distance	$\sqrt{\sum_{l=1}^{200}{\left(WE_{w}^{\left(m,i\right)}\left[l\right]\;+\;WE_{n}^{\left(n,j\right)}\left[l\right]\right)}^{2}}$
Cosine Distance	$\frac{\sum_{l=1}^{200}WE_{w}^{\left(m,i\right)}\left[l\right]\;\times \;WE_{n}^{\left(n,j\right)}\left[l\right]}{\sqrt{\sum_{l=1}^{200}WE_{w}^{\left(m,i\right)}{\left[l\right]}^{2}}\;\times \;\sqrt{\sum_{l=1}^{200}WE_{n}^{\left(n,j\right)}{\left[l\right]}^{2}}}$
Manhattan Distance	$\sum_{l=1}^{200}\left|WE_{w}^{\left(m,i\right)}\left[l\right]\;-\;WE_{n}^{\left(n,j\right)}\left[l\right]\right|$
Chebyshev Distance	$\underset{k\to \infty }{\lim}{\left(\sum_{l=1}^{200}\left|WE_{w}^{\left(m,i\right)}\left[l\right]\;-\;WE_{n}^{\left(n,j\right)}\left[l\right]\right|\right)}^{\frac{1}{k}}$
Standardized Euclidean Distance	$\sqrt{\sum_{l=1}^{200}{\left(\frac{WE_{w}^{\left(m,i\right)}\left[l\right]+WE_{n}^{\left(n,j\right)}\left[l\right]}{s\left[l\right]}\right)}^{2}}$ where *s*[*l*] is standard deviation

**Table 2:** The Dataset of cross-platform transportation in Qingdao.

	Training Dataset (about 2 Years)			Testing Dataset (7 Days)			Case Study Dataset (1 Day)		
	Text	Authors	Roads	Text	Authors	Roads	Text	Authors	Roads
News Articles	301,684	-	132	2369	-	37	334	-	12
Weibo Posts	839,587	271,260	147	4072	2600	49	581	428	25

**Table 3:** Most similar words for traffic congestion and traffic accident ranked by transportation word2vec.

Traffic Words	Similar Words	Similarity	Traffic Words	Similar Words	Similarity
交通拥堵 Traffic congestion	交通拥挤 Heavy traffic	83.47%	交通事故 Traffic accident	交通事件 Traffic incident	82.19%
	交通阻塞 Traffic jam	83.46%		车祸 Car accident	69.77%
	堵车 Caught in traffic	75.28%		伤亡事故 Fatality accident	62.43%
	塞车 Stuck in traffic	60.27%		碰撞 Traffic crash	60.86%
	滞留 Traffic blocking	59.45%		重大事故 Major accident	59.33%

**Table 4:** The topic detection results from Weibo and News.

Road	$T_{n}$	$T_{w}$	Type
抚顺路 Fushun Road	抚顺路 施工现场 堵车 交叉路口 缓慢… Fushun Road, construction site, traffic jams, intersection, slow moving…	交通广播 抚顺路 一动不动 堵死 附近 高峰时段 疏通 水泄不通… Traffic broadcast, Fushun Road, blocked, slow moving, nearby, peak hours, dispersion, crowd and jam…	拥堵 Traffic Jam
银川西路 Yinchuan WestRoad	银川西路 会场 一段路 规划 答复 拥堵… Yinchuan WestRoad, theater, road section, city planning, response, traffic jams…	信号灯 失灵十字路口 怎么回事 东向西 吐槽 为何… Traffic signals, not work, crossroads, what happened, east to west, complain, reason…	投诉 Traffic Complaint
人民路 RenMin Road	人民路 交警 民警 男子 驾驶 撞墙 双腿 受伤… RenMin Road, traffic police, police officer, man, car driving, hit the wall, legs, injured…	人民路 救援 现场 不慎 情况危急 求助 车底… RenMin Road, rescue, accident scene, critical situation, help, car bottom…	事故 Traffic Accident

**Table 5:** Average and standard deviation of topic distances for different roads.

Distance Measure (R=)	Average and Standard Deviation	人民路 RenMin Road	伊春路 YiChun Road	山东路 ShanDong Road	延吉路 JiYan Road	延安三路Yan’ An San Road	抚顺路 FuShun Road	敦化路 AnHua Road	登州路 DengZhou Road	银川西路 YinChuan Xi Road	鞍山路 AnShan Road
Euclidean	$\mu_{R}\left(T_{w}^{\left(i\right)},T_{n}^{\left(j\right)}\right)$	0.896	1.088	1.03	1.09	0.973	0.986	1.101	0.96	1.083	0.916
	$\sigma_{R}^{*}\left(T_{w}^{\left(i\right)},T_{n}^{\left(j\right)}\right)$	0.07278									
Cosine	$\mu_{R}\left(T_{w}^{\left(i\right)},T_{n}^{\left(j\right)}\right)$	0.0031	0.0041	0.0033	0.0036	0.0029	0.0032	0.0071	0.0034	0.0036	0.0029
	$\sigma_{R}^{*}\left(T_{w}^{\left(i\right)},T_{n}^{\left(j\right)}\right)$	0.00117									
Manhattan	$\mu_{R}\left(T_{w}^{\left(i\right)},T_{n}^{\left(j\right)}\right)$	10.15	12.33	11.31	12.28	10.97	11.12	12.36	10.81	12.21	10.32
	$\sigma_{R}^{*}\left(T_{w}^{\left(i\right)},T_{n}^{\left(j\right)}\right)$	0.8176									
Chebyshev	$\mu_{R}\left(T_{w}^{\left(i\right)},T_{n}^{\left(j\right)}\right)$	0.169	0.206	0.191	0.207	0.185	0.189	0.207	0.181	0.208	0.174
	$\sigma_{R}^{*}\left(T_{w}^{\left(i\right)},T_{n}^{\left(j\right)}\right)$	0.01386									
Weighted Euclidean	$\mu_{R}\left(T_{w}^{\left(i\right)},T_{n}^{\left(j\right)}\right)$	19.78	19.99	19.68	19.54	19.91	19.6	19.54	20	19.91	20
	$\sigma_{R}^{*}\left(T_{w}^{\left(i\right)},T_{n}^{\left(j\right)}\right)$	0.17455									

**Table 6:** Performance comparison of Baseline and the proposed model.

	Hits	Miss	False Alarm	Precision	Recall	F_1_ Score
Baseline Model	50	37	4	92.6%	57.4%	70.9%
The Proposed Model	74	13	7	91.4%	85.1%	88.1%

**Table 7:** Top 3 hottest fused traffic events in Qingdao on 4 August 2017.

Target Road	Relevant Locations	Traffic Words	Traffic Causal Words	Other Words
金沙滩路 Gold Beach Road	黄岛区 啤酒城 隧道 Huangdao District, Beer Square, Cross-sea Tunnel.	缓慢 交通拥堵 高峰 客流 车流量 停车 交警 Slow, traffic jam, rush hour, passenger flow, traffic flow, parking, traffic polices	啤酒节 开幕式 开园 Beer festival, opening ceremony, park opening	黄晓明 明星 直播 Xiaoming Huang, Superstar, live show
同安路 Tong An Road	国信体育场 银川东路 劲松七路 海尔路 Guoxin Stadium, Yin Chuan Xi Road, Jin Song Qi Road, Hai Er Road	绕行 高峰 慢行 管制 拥堵 停车 调流 交通广播 预案 Passing round, rush hour, slow, traffic control, traffic jam, parking, traffic flow regulation, traffic broadcast, Traffic plan.	五月天 演唱会 Mayday singer group, concert	门票 五迷 提示 Tickets, fans, notice
延安三路 Yan An San Road	石油大厦 Petroleum Building	交通拥堵 交警 交通广播 道路封锁 车辆疏导 疏散 Traffic jam, traffic policies, traffic broadcast, road closed, traffic dispersion, evacuation	火情 火警 起火 扑灭 Fire situation, fire alarm, catch fire, Fire extinguished	伤亡 电梯井 Casualties, elevator hoist way

## Appendix A. Table of Variables

**Symbol**	**Description**	**Data Structure**	**Supporting Process**
*T*	Number of Weibo posts	Int *T*	*w*-LDA
*U*	Number of Weibo users	Int *U*	
*K*	Number of topics	Int *K*	
*V*	Number of words in the vocabulary	Int *V*	
*V*	Number of uers profiles	Int *V*	
*N_p_*	Number of words in p-th user profile	Int N[*P*]	
α	K-dimensional prior weight vectors of topics in a document,	Float a[*K*]	
β	V-dimensional vector prior weight of words in a topic	Float b[*V*]	
$\varphi_{z}$	V-dimensional vector of probabilities, represents distribution of words in topic z	Double phi [*Z*][*V*]	
$\vartheta_{p}$	K-dimensional vector of probabilities, represents distribution of topics in user profile p	Double theta [*P*][*K*]	
$z_{i}$	Identity of current topic of word $w_{i}$ in user profile $p_{i}$	Int *1…K*	
$w_{i}$	Identity of current word in user profile $p_{i}$	Int *1…V*	
$p_{i}$	Identity of current user profiles	Int *1…P*	
$n_{i,j}^{\left(p_{i}\right)}$	Document-Topic matrix, the number of times topic j has been assigned to words in user profile $p_{i}$.	int npt [*P*][*K*]	
$n_{i,j}^{\left(w_{i}\right)}$	Topic-Word matrix, Number of times that word $w_{i}$ has been assigned to topic j	int ntw [*K*][*V*]	
***W_i_***	Identity of current word vector (200 dimensions) trained by traffic word2vec	Double [200]	*Similarity measure*
$T_{w}^{\left(i=1\ldots K\right)}$	Identity of current topic word cluster detected from Weibo	Double tw[*K*]	
$T_{n}^{\left(j=1\ldots K\right)}$	Identity of current topic word cluster detected from News	Double tn[*K*]	
$WE_{w}^{\left(m,i\right)}$	Word embedding- cluster tensor, identity of the current word embedding in i-th cluster detected from Weibo	Double cew[*K*] [***W_i_***]	
$WE_{n}^{\left(n,j\right)}$	Word embedding- cluster tensor, identity of the current word embedding in i-th cluster detected from News	Double cen [*K*] [***W_i_***]	
$dis_{R}\left(WE_{w}^{\left(m,i\right)},WE_{n}^{\left(n,j\right)}\right)$	Words similarity, measure the similarity between word embedding $WE_{w}^{\left(m,i\right)}$ and $WE_{n}^{\left(n,j\right)}$	Double wd	
$D_{R}\left(\;T_{w}^{\left(i\right)},\;T_{n}^{\left(j\right)}\right)$	Topic similarity matrix, measure the distances between each words in the given topic cluster $T_{w}^{\left(i\right)}$ and $T_{n}^{\left(j\right)}$	Double td[K][K]	*Event fusion*
$\mu_{R}\left(T_{w}^{\left(i\right)},T_{n}^{\left(j\right)}\right)$	Average shortest distance between $T_{w}^{\left(i\right)}$ and $T_{n}^{\left(j\right)}$	Double atd	
$\mu_{R}^{*}\left(T_{w}^{\left(i\right)},T_{n}^{\left(j\right)}\right)$	Normalized average shortest distance between $T_{w}^{\left(i\right)}$ and $T_{n}^{\left(j\right)}$	Double natd	
$\sigma_{R}^{*}\left(T_{w}^{\left(i\right)},T_{n}^{\left(j\right)}\right)$	The standard deviation of normalized topic distances	Double sd	
